# Atmospheric Boundary Layer Wind Profile Estimation Using Neural Networks Applied to Lidar Measurements

**DOI:** 10.3390/s21113659

**Published:** 2021-05-24

**Authors:** Adrián García-Gutiérrez, Diego Domínguez, Deibi López, Jesús Gonzalo

**Affiliations:** Aerospace Engineering Area, Universidad de León, 24071 León, Spain; diego.dominguez@unileon.es (D.D.); deibi.lopez@unileon.es (D.L.); jgonzalo@unileon.es (J.G.)

**Keywords:** neural network, wind vertical profile, lidar, atmospheric boundary layer

## Abstract

This paper introduces a new methodology for estimating the wind profile within the ABL (Atmospheric Boundary Layer) using a neural network and a single-point near-ground measurement. An important advantage of this solution when compared with others available in the literature is that it only requires near surface measurements for the prognosis once the neural network is trained. Another advantage is that it can be used to study the wind profile temporal evolution. This work uses data collected by a lidar sensor located at the Universidad de León (Spain). The neural network best configuration was determined using sensibility analyses. The result is a multilayer perceptron with three layers for each altitude: the input layer has six nodes for the last three measurements, the second has 128 nodes and the third consists of two nodes that provide *u* and *v*. The proposed method has better performance than traditional methods. The obtained wind profile information obtained is useful for multiple applications, such as preliminary calculations of the wind resource or CFD models.

## 1. Introduction

The atmospheric boundary layer (ABL) is the region of the atmosphere that defines the transition between the upper geostrophic winds and the static air layers in contact with the earth’s surface. Most human activities take place in this region, thus our knowledge and understanding of the ABL is critical for many important applications. Some of them can be found in wind energy predictions [1,2], the construction and insurance industries [3], simulations of pollutant and toxic gas release [4,5], and even Air Traffic Management (ATM) automation [6,7]. Although these activities could be influenced by different atmospheric parameters, wind speed and direction are the most critical ones and have attracted most of the attention. However, measuring and/or predicting them is not an easy task.

When a precise knowledge of the vertical wind speed profile is needed, in-situ measurement procedures are required. There is a broad range of available technologies (from well-known meteorological towers or light detection and ranging—lidar [8]—to non-conventional techniques like bubble tracking [9,10]) with different capabilities and limitations.

Thus, different research efforts have been made to produce accurate and reliable ABL wind profiles avoiding the need of complex and expensive measurement systems. Among the different studies, two main approaches can be found: physical and statistical. The former methods use atmospheric weather prediction models [11,12] while the latter methods rely on linear time-series models [13,14], wavelet transforms [15], artificial neural networks [16,17] or hybrid methods [18,19] that unify the several techniques previously mentioned.

However, none of the approaches presented until now is totally satisfactory, as most of the times the vertical profile has not been studied or, when it is considered, its temporal evolution is not addressed [20,21]. This paper presents a novel methodology for the prognosis of complete ABL wind profiles that only requires a limited set of surface observations. The proposed approach comprises the use of a neural network that will be trained with 60 days of lidar wind profile measurements (wind speed and direction). Lidar sensors offer unique advantages for wind profiling [22] and are the preferred solution for high-end applications. Nevertheless, they are really expensive instruments that make them unaffordable for most applications. This is especially true when considering that wind profiles are highly site-dependent thus, the characterization of the ABL over a certain area (e.g., for the construction of a wind farm) would require the installation of multiple lidar stations at several different locations.

This paper presents a more economical alternative. As it will be detailed, the developed neural network only requires 60 (non-consecutive) training days of profile measurements per year and location. Such a model makes it possible to monitor up to 6 different locations during a year with a single lidar station. Once the neural network has been trained for a certain location, the lidar can be replaced by a cheaper instrument that takes local wind measurements (e.g., ultrasonic anemometers which are 50 times cheaper). The neural network will then use the data series provided by the anemometer to generate a full ABL wind profile.

Although there are previous studies that use artificial neural networks (ANNs) to interpolate wind profiles, they present several differences with the methodology proposed in this one. Vassallo, Krishnamurthy and Fernando [23] measurements at multiple heights, not just the lowest, for doing the interpolation. So, for example, in order to estimate the wind at 120 m, they used the values observed at 100, 80, 60 and 40 m AGL (above ground level). Thanks to that, the accuracy that their ANN can achieve is between 65% and 53% better than that obtained by a log-law or power law vertical extrapolations.

Turkan et al. [24] analyzed seven ML algorithms and studied its accuracy in extrapolating the wind resource from 10 m to 30 m AGL at a wind farm in Turkey. Between the different methods, the support vector regression, the multilayer perceptron and the Random Tree obtained good performance. They used training data collected during 11 months to predict the values during December, so the lidar would not be able to monitor several locations at the same time.

Mohandes and Rehman [25] also use different heights (i.e., the values at 10, 20, 30 and 40 m are required to compute the value at 120 m). Moreover, they only consider wind magnitude, so the wind direction is not tackled. At 120 m height, the mean absolute percent error achieved is 9.65%.

More recently, Bodini and Optis [26] used a random forest algorithm to extrapolate the 30-min average wind speed at 143 m AGL using the values measured at 65 m AGL and other physical parameters such as the turbulent kinetic energy and the Obkuhov length measured at 4 m AGL. They found that their method improvement in mean absolute error was 28% and 23% over the power law and logarithmic profile. However, they need to use a lidar to obtained the wind value at 65 m AGL, so no cost reduction is obtained.

As it can be seen, previous studies have different objectives from the present one. Here, the main objective is to establish a methodology which could reduce the number of lidar required to monitored several locations. So, in the present study, the prediction uses only one near ground sensor. Additionally, the training dataset required by the ANN should be relatively small: to monitored one location all the year, it is only required to have the lidar at that location 1–2 months.

This paper is organized as follows. Section 2 describes the artificial neural network implementation, as well as the acquisition method and the statistical properties of the wind datasets. Section 3 presents different sensitivity analysis performed in order to find the best configuration for the neural network. Once this configuration is defined, the simulation results are compared against real data using different scoring rules in Section 4. Finally, the conclusions are summarized in Section 5.

## 2. Materials and Methods

Two elements will be needed to successfully perform the prognosis of the ABL wind profile relying on just near-surface observations. On the one hand the neural network that shall extrapolate available data and, on the other hand, the required set of observations to train it. Both are now presented.

### 2.1. Multi-Hidden Layer Neural Network

Multi-hidden layer neural networks (MHL-NN), also known as deep neural networks, are widely used in different kind of tasks such as process monitoring, fault diagnosis and, more relevant for this work, atmospheric modeling [27]. MHL-NN process works as a black box, modeling the output variables as the results of the inputs through a systematic method. Each of the network nodes are called neurons and are grouped in layers. Each layer of *l* neurons applies an activation function over the weighted sum of its *p* inputs following the equation [28]:

(1) g=σ(Ws+b),

in which *s* is the input vector, W is a matrix of dimensions l×p and $b\in R^{l}$. W and *b* represent the training parameters. There are different choices for the mapping functions [29] such as the sigmoid function $\sigma \left(x\right)=\frac{1}{1+e^{-x}}$, the hyperbolic tangent function $\sigma \left(x\right)=\frac{e^{x}-e^{-x}}{e^{x}+e^{-x}}$ or the rectified linear unit σ(x)=max(x,0) being the variable x∈R.

The network is a stack of these neurons, from the input layer to the output one. For the first layer, Equation (1) takes the form:

(2) $$g_{1}=\sigma \left(W_{1}X+b_{1}\right),$$

given a fixed matrix $W_{1}\in R^{l_{i}\times n}$, vector $b_{1}\in R^{l_{i}}$ and $X\in R^{n}$ the input vector. Here, *n* denotes the number of inputs of the neural network. Then, for the subsequent hidden layers, its output vector can be found from the previous layer as:

(3) $$g_{i}=\sigma \left(W_{i}h_{i-1}+b_{i}\right),$$

until the output layer is finally reached:

(4) $$\hat{u}=W_{o}h_{k}+b_{0},$$

in which $\hat{u}$ is the final prediction.

The scale of inputs and outputs used to train the model is a relevant factor [30]. To prevent a slow or unstable learning process the standardization of each of the input and output variables $\hat{x}$ is done according to the formula:

(5) $$\hat{x}=\frac{x-\bar{x}}{\sigma_{x}},$$

where $\bar{x}$ is the mean of *x* in the training set and $\sigma_{x}$ is its standard deviation.

After the neural network architecture is determined, the training phase can be started. In order to train the neural network, the weights $\left\{W_{0}\cdots W_{k}\right\}$ and $b=\{b_{0},\cdots b_{k}\}$ need to be modified such that a cost function is minimized. Usually, this cost function is defined as the sum of squares of the network errors MSE:

(6) $$MSE=\frac{1}{n}\sum_{i=1}^{n}{\left({\hat{u}}_{i}-u_{i}\right)}^{2}.$$

To obtain the optimal parameters, the cost function MSE is minimized by using back propagation algorithms for the training dataset. Between the different options, the Bayesian regularization [31], the scaled conjugate gradient method [32], and the Levenberg–Marquardt learning algorithms [33] stand out. The main advantage of using neural networks is their capability to automatically find an optimal way to combine the input variables through a data-driven approach. In order to avoid over-fitting of the training dataset an early stopping method is applied based on the validation loss [34].

For the present study, the algorithm was implemented using the open-source neural-network library Keras [35] written in Python. Using Keras terminology, the neural networks described in this section, also known as Feed Forward Networks (FFNNs), could be built as a sequential model, i.e., plain stack of layers where each layer was of type “Dense”. As the backend engine of Keras, the library TensorFlow [36] was selected. More information about the library can be found in [35].

### 2.2. Wind Data

For any neural network application, the quality and availability of training data sets are critical. Additionally, those data will be needed for the validation and performance assessment of the neural network once it has been trained. In our problem, the wind data (magnitude and direction) of the ABL profile were needed for the heights of interest and a period of time large enough.

**Figure 1:**
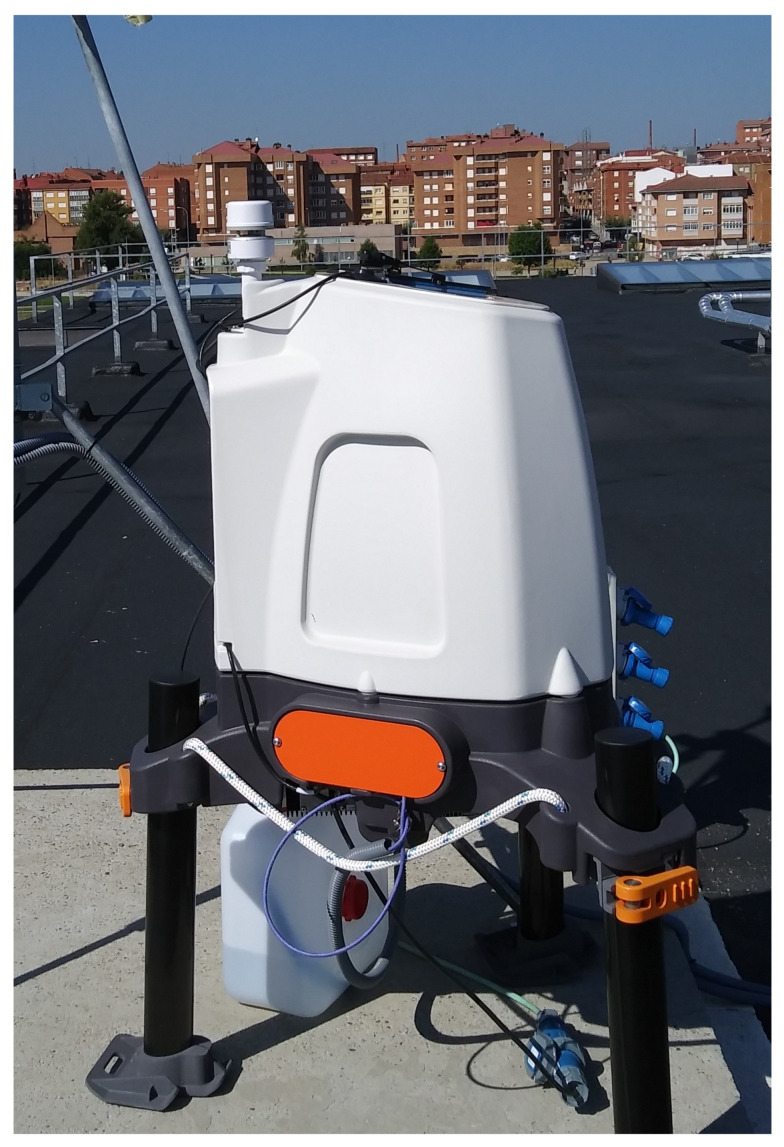
ZephIR 300 wind lidar.

The dataset used in this paper was the 10-min averaged wind speed and direction measurements produced by a wind lidar station located in a sub-urban area in León (Spain) during the period 2018–2020 (more than 25,300 samples). Such a kind of long term and high-quality measurements is really difficult to obtain, as the required equipment is very expensive and highly demanded. Wind lidars determine the wind velocity components for a specific range of altitudes using pulsed laser light and measuring the reflected pulses with a sensor. The model used for the validations is the ZephIR300 lidar (Figure 1) validated in previous works [37]. ZephIR300 is specialized in the optimization of eolic parks and its main characteristics can be found in Table 1.

**Figure 2:**
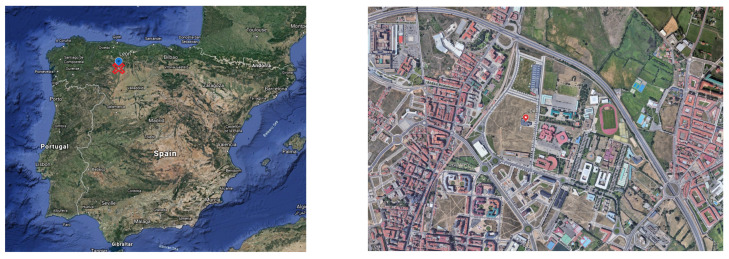
Localization of León (Spain) and the lidar position in an urban area within the campus of the Universidad de León.

The lidar was similar to the one used by Kent et al. [20], it operated in Doppler Beam Swinging (DBS) mode, whereby the measured Doppler shift between transmitted and returned pulses provided horizontal wind speed and direction. The system was configured to take measurements at eleven different altitude levels between 30 and 300 m AGL: 30 m, 50 m, 65 m, 90 m, 120 m, 150 m, 180 m, 220 m, 250 m, 280 m, 300 m. The lidar was located at the University of León campus with coordinates 42${}^{{}^{\circ}}$36${}^{\prime }$ 54.1${}^{\prime \prime }$ N 5${}^{{}^{\circ}}$33${}^{\prime }$49.5${}^{\prime \prime }$ W (northwest of Spain as can be seen in Figure 2). Following the Davenport roughness classification, the roughness length of the terrain should be close to 2 m, typical of regions with mixture of low-rise and high-rise buildings [38]. The exact value was calculated from measurements in Section 3.

A brief statistical analysis of the data gathered during the sampling period was done in order to study the spatial and temporal characteristics of the wind profile. This kind of correlation analysis has been done traditionally with the wind speed modulus (V) and direction (β), however, we also performed an additional study for the north (*u*) and west (*v*) wind components aiming to look for a better correlation. Both presentations of wind characteristics were related as:

(7) $$\begin{array}{c} V=\sqrt{u^{2}+v^{2}}, \end{array}$$

(8) $$\begin{array}{c} \beta =\arctan2(u,v). \end{array}$$

**Figure 3:**
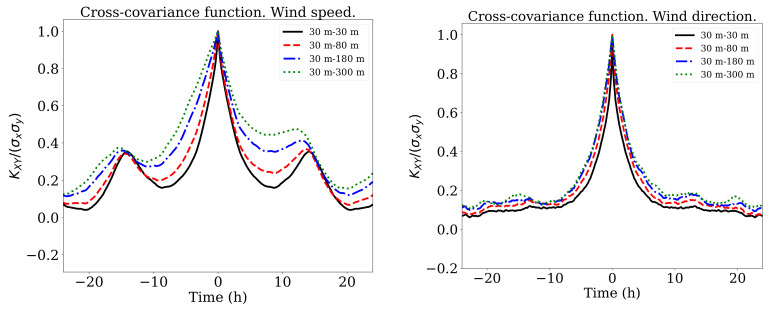
Cross covariance function of V (**left**) and β (**right**) between different altitudes.

**Figure 4:**
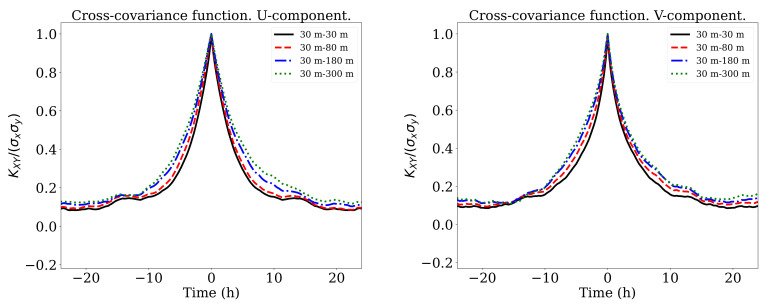
Cross covariance function of *u* (**left**) and *v* (**right**) between different altitudes.

From previous studies, Velo et al. [39] determined that the linear correlation was a critical criterion to determine the suitability of the dataset for the neural network. Thus, the temporal cross-covariance between different heights was computed as:

(9) $$K_{xy}\left(\tau \right)=E\left[\left(x-\bar{x}\right)\left[t+\tau \right]{\left(y-\bar{y}\right)}^{T}\left[t\right]\right],$$

in which *x* and *y* represents the measurements obtained at two different height, *t* is the time and τ the time lag. E[·] and ${\left[\cdot \right]}^{T}$ denote respectively the expected value and the transpose of a given random vector. The results are shown in Figure 3 for V and β. The most interesting aspect of these graphs was the two peaks in the wind speed correlation at ±15 h which seemed to suggest a dependence between diurnal and night values. These peaks were less marked in the *u*,*v*-cases as is shown in Figure 4.

**Figure 5:**
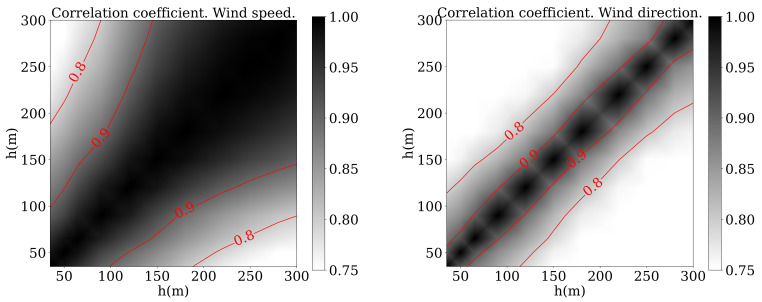
Pearson correlation coefficient of V (**left**) and β (**right**) between different altitudes.

**Figure 6:**
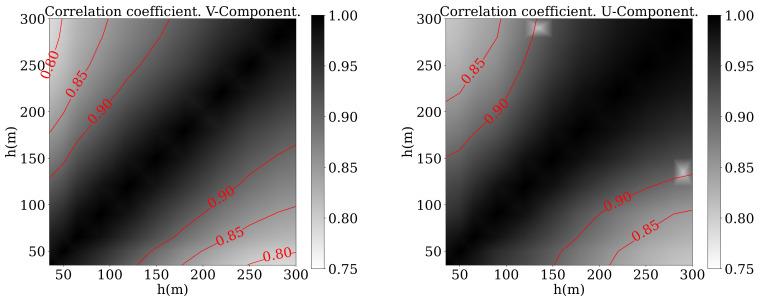
Pearson correlation coefficient of *u* (**left**) and *v* (**right**) between different altitudes.

Another relevant parameter that has been traditionally used to study the correlation [39] is the Pearson correlation coefficient, defined as:

(10) $$CC=\frac{\sum_{i=1}^{N}\left(x_{i}-\bar{x}\right)\left(y_{i}-\bar{y}\right)}{\sqrt{\sum_{i=1}^{N}{\left(x_{i}-\bar{x}\right)}^{2}}\sqrt{\sum_{i=1}^{N}{\left(y_{i}-\bar{y}\right)}^{2}}}$$

where $x_{i}$ and $y_{i}$ are the variable values at two different altitudes and $\bar{x}$, $\bar{y}$ are the average values for a period of time. Figure 5 shows the results for V and β while Figure 6 shows the results for *u* and *v*. In those figures black color denotes a very high correlation between the heights indicated in horizontal and vertical axes. The correlation is 1 for the diagonal, as it indicates the relationship between a certain height and itself. Measurements presented a higher correlation with those taken at a closer location and decays when compared to values measured higher or lower in the profile. That explains the white colour close to the corners. What is striking about these figures is that, in the case of the wind direction, the correlation decayed faster than in the other three cases.

The temporal and spatial correlations suggested that useful results could be obtained by means of neural networks. The capabilities of these networks will be tackled in the next sections.

## 3. Optimal Configuration

Once the statistical analysis of the data indicated that the selected variables can be used with a neural network, it was time to look for the best configuration of the tool e.g., training patterns, learning algorithm, input data, etc.

**Figure 7:**
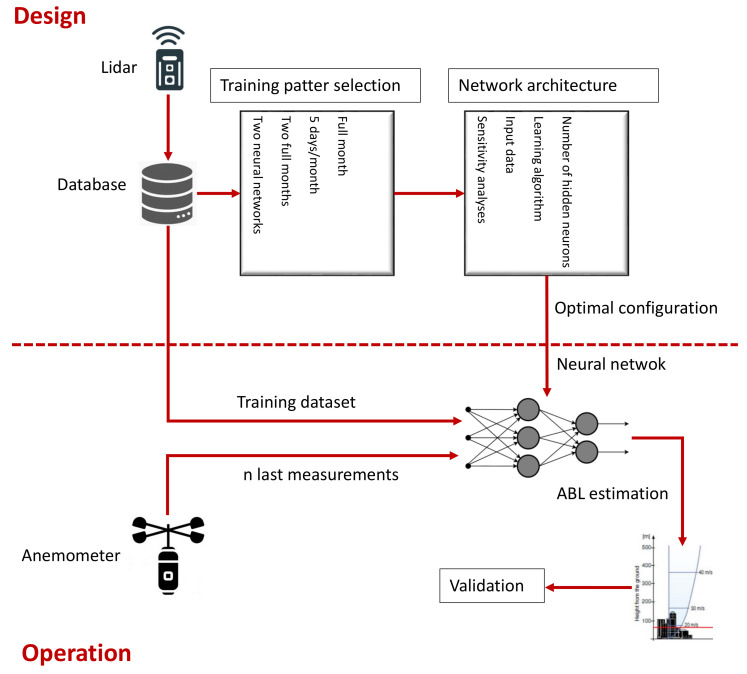
Research flowchart.

As it can be seen in Figure 7, once this optimal design was found, the lidar would only be required for a relatively short period of time to train the ANN. Then, using just near ground measurements, the ANN could estimate the complete wind profile without requiring a expensive lidar sensor anymore.

### 3.1. Numbers and Definition of the Training Patterns

At this point, it is worth remembering that the objective was to estimate the vertical wind profile as function of the wind velocity near the ground. In order to achieve that using MHL-NN, we could use as input/output variables the directional components of the wind—*u* and *v*—or the wind speed V and direction β.

Although related works used the wind speed and direction [39], Figure 5 and Figure 6 show how the correlation was higher in the case of *u* and *v*, so it is not clear which option should be the best. In addition, the number of patterns that had to be used to train the network was also unknown a priori. As an activation function, the sigmoid function seemed to be the best option for the wind prediction [40].

For determining which is the optimal configuration, the wind at an altitude of 180 m was simulated using the measurements from 30 m. To compare the results, the scoring rules used [41] were the root mean square error (RMSE), the mean absolute error (MAE) and the mean absolute percentage error (MAPE):

(11) $$\begin{array}{c} RMSE=\sqrt{\frac{\sum_{t=1}^{n}{\left(y_{t}-{\hat{y}}_{t}\right)}^{2}}{n}}, \end{array}$$

(12) $$\begin{array}{c} MAE=\frac{1}{n}\sum_{t=1}^{n}\left|y_{t}-{\hat{y}}_{t}\right|, \end{array}$$

(13) $$\begin{array}{c} MAPE=\frac{1}{n}\sum_{t=1}^{n}\left|\frac{y_{t}-{\hat{y}}_{t}}{y_{t}}\right|\times 100\%, \end{array}$$

where $y_{t}$ is the actual variable (*u*, *v*, V or β) and ${\hat{y}}_{t}$ is the simulated value. *n* is the number of samples. As characteristic wind speed, it is defined the root mean square (RMS) as:

(14) $$V\;RMS=\sqrt{\frac{\sum_{t=1}^{n}V_{t}^{2}}{n}}.$$

This first sensibility analysis was done with a neural network of three layers: the input layer, with six neurons that took the last three 10-min averaged measurements values (at *h* = 30 m); the hidden layer, with 64 nodes; and the output layer, with two neurons that gave us the wind variables at 180 m. The optimization algorithm used was the RMSProp [42].

The results are shown in Table 2 (using *u* and *v* as inputs) and Table 3 (using V and β). The yellow color, from now on, highlights the best estimation in each column. The cases tested were: (1) using as training data a full month of the year (January, February, August or September); (2) using 5 days of each month (1st–5th or 6th–10th); (3) using 2 full months (January and February or August and September); (4) using two different neural networks, each one for 6 months and trained with a full month data per network (using the months of January and September or February and August).

According to the values shown, the best configuration was option (2) that trained the network using 5 days of each month of the year. That was true for both the RMSE and the MAE. This conclusion is in line with previous works as the result obtained in [39]. The options (3) and (4) also achieved good results, with errors slightly higher than those obtained by (2). Due to these small differences, they may be recommended in other circumstances. On the other hand, option (1) seemed to rely heavily on the month chosen for simulation. For example, if the month was January, the uRMSE reached a value of 1.95 m/s which was 12% higher than in any of the other options. Similar behavior could be found in the other variables.

It may be seen that moving a lidar every 5 days is not pragmatic. That depends on different aspects such as the lidar model or the geographic conditions. For example, the lidar used in this study weighed 55 kg and could be connected via mobile networks. So, no technical staff was required to reconfigure it, and it could be easily move by two workers using the handles (as can be seen in Figure 2). Nevertheless, it might be more practical to do 2-month training (Jan and Aug or Feb and Sep), thereby reducing the amount of time required to move the lidar. The performance in that case was only slightly penalized as it is shown in Table 2.

Although previous work in the literature has made use of a (V,β) definition of the wind vector, the approach suggested in this work of using vector components (*u*, *v*) demonstrated a better performance in terms of estimating *u*, *v* and β. The opposite was true for the wind speed. Even though it would depend on each case, we considered that, for most of the applications, the improvements achieved by (*u*, *v*) were worthwhile. For example, the uRMSE decreased from 2.8 m/s to 1.65–1.67 m/s which was a reduction of 40%. A relevant improvement could be also found in *v* (16%) and β (34%). On the other hand, the value of VRMSE increased by 4%.

Therefore, the configuration used in this study consisted of using the variables *u* and *v* as inputs and trained the network with 5 consecutive days of each month of the year.

### 3.2. Selection of the Number of Hidden Neurons, Learning Algorithm and Input Data

Once the training patterns were chosen, the optimal number of neurons and the best learning algorithm for this case was the next step to solve.

The wind speed at an altitude of 180 m was simulated using, again, measurements taken at 30 m. Firstly, different architectures of the neural network were used, with one or two hidden layers. As can be seen from the results of the score rules presented in Table 4, it seemed that using two layers instead of one did not achieve better results. Additionally, neural networks with more than 128 nodes did not show a relevant increment in accuracy.

Although the objective was to estimate the vertical profile in real time, the neural network may take advantage of previous wind measurements. Every 10 min a new reading was available. As it is shown in Figure 3, the correlation was relatively high for temporal windows of 3 h with peaks at ±12 h. Six different cases were analyzed using: (1) the last three measurement (30-min); (2) the last six measurements (1-h); (3) only the last measurement (10-min); (4) three measurements equispaced during the last 3 h; (5) three measurements equispaced during the last 6 h, and (6) three measurements equispaced during the last 12 h. In every case, each of the measurements used was a input node of the neural network. It is apparent from Table 5 that no advantage could be found in including more than the three last measurements. If we included only one measurement, the errors grew between 2.5% (uRMSE) and 5.3% (VRMS). It is interesting to note how option (6) achieved good results predicting the variable V. That can be related to the temporal correlation showed in Figure 3 and its peaks at ±12 h.

Then, different optimization algorithms were compared to find the optimal. Between the different options, the following were tested: RMSprop [42], SGD [43], the stochastic solver Adam [44], Nadam [45] and Ftrl [46]. The results, as shown in Table 6, indicated that the RMSprop slightly outperformed the other algorithms. It was the best option for V and β and only the Nadam algorithm was better in terms of uMAE. However, it is worth noting that the differences found between the algorithms were very small, lower than 3%.

Finally, the sensibility of the batch size, learning rate, hyperparameters, optimizer algorithm and activation function were analyzed using the Keras Tuner [47]. As the optimal configuration of the ANN depended on *h*, different designs were found for each height; however, no substantial improvements were obtained (a reduction <1% in the MAE). Due to this, for simplicity’s sake, the same baseline configuration was used for all the levels.

## 4. Results of the Wind Profile Prognosis

After the sensibility analysis performed in the previous subsections, the neural network configurations were as follows:

- Six nodes in the input layer which took the 10-min average values of *u* and *v* from the last three measurements at h=30 m.
- The training data corresponded to 5 days of each month.
- 128 nodes in the hidden layer, using the sigmoid function as activation function.
- Two output neurons which gave the values of *u* and *v* for a certain altitude.
- There was one neural network for each of the altitudes.

At this point, the neural network was ready to be used to perform the wind profile prognosis. In this section, the performance of the neural network is analyzed and compared with other traditional methods. It will also be studied how that performance can be improved by including daily and hourly information.

### 4.1. Global Performance

Table 7 shows some outstanding characteristics of the results obtained. The correlation coefficients between the real wind speeds and those simulated by the neural network were higher than 0.8 for all the cases. Furthermore, at the lower altitudes (below 180 m) the correlation was even higher than 0.9, which represented a remarkably high value (notice that correlation was, for example, 0.96 at 90 m; that was three times the measurement altitude).

**Figure 8:**
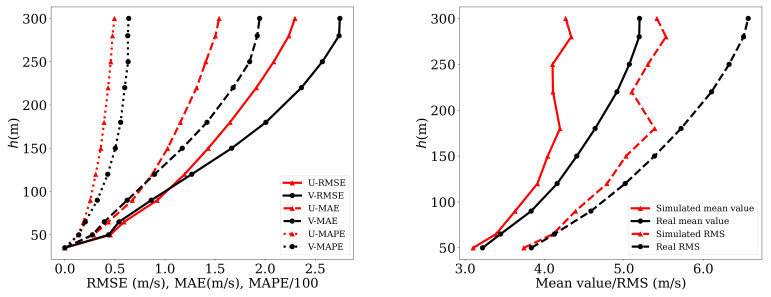
Analysis of the accuracy for the estimated wind profiles. **Left image**: Values of the different score rules at every height. **Right image**: Simulated and real mean and RMS velocity values at every height.

Figure 8 (left) shows the vertical profile of the different score rules. In the right image, the mean velocity and its RMS value for different altitudes are shown. The difference between both RMS curves showed again that prognosis capability was notably good up to an height of 180 m. Over that the capabilities of the tool could be still considered as useful but its accuracy notably deteriorated.

**Figure 9:**
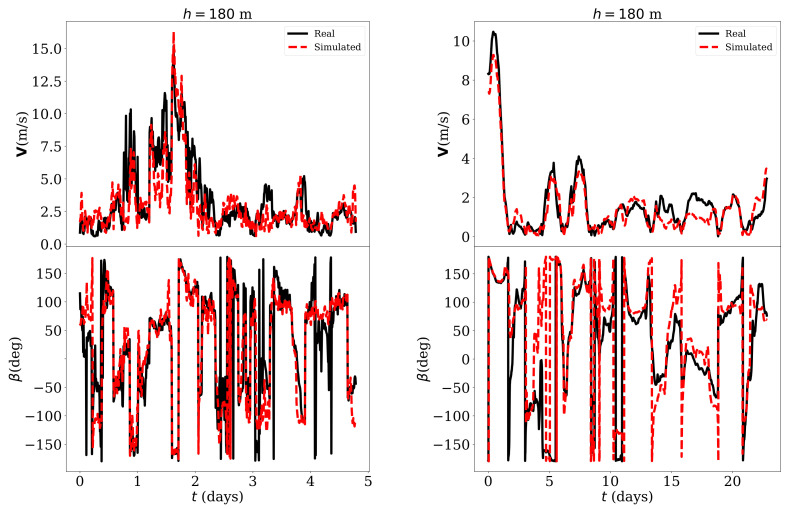
Simulated and real temporal series of V and β for h=180 m. **Left image**: 10-min averaged values, 1st-5th February 2019. **Right image**: Hourly averaged values, February 2019.

**Figure 10:**
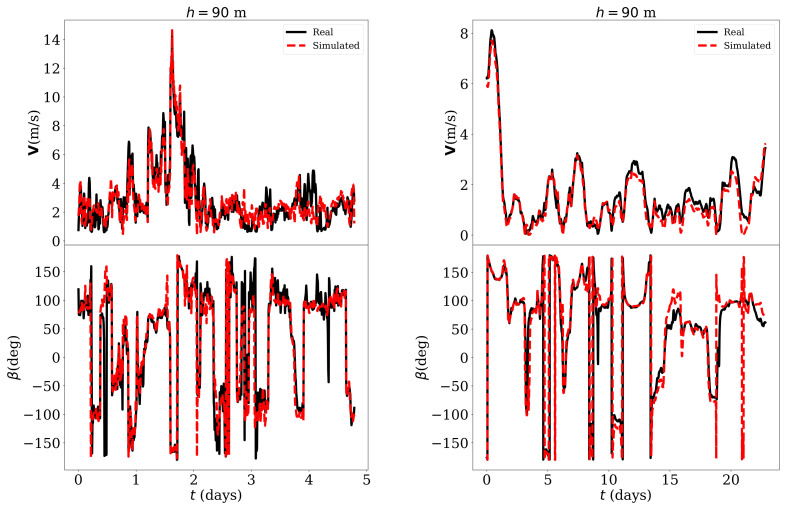
Simulated and real temporal series of of V and β for h=90 m. **Left image**: 10-min averaged values, 1st-5th February 2019. **Right image**: Hourly averaged values, February 2019.

The good agreement between the simulation results and the measurements envisaged thanks to the high value of the Pearson coefficient could also be verified directly by observing the simulated and real temporal series. For example, Figure 9 shows—for h=180 m—the 10-min temporal series of V and β—values reconstructed with the estimated variables *u* and *v* using Equation (7) and (8)—for 5 days in February, 2019 which were not part of the training data. In the same figure, the hourly-averaged temporal series of the same variable for the whole month was also compared. The results for h=90 m are shown in Figure 10 in which a lower estimation error could be noted.

For h=90m and in the case of 10-min averaged values, the errors were VRMS=0.32 m/s, VMAE=0.27 m/s, VMAPE=15.61%, $\beta \;RMS=28.6^{{}^{\circ}}$, $\beta \;MAE=14.3^{{}^{\circ}}$ while, in the case of the hourly averaged values, the values were VRMS=0.88 m/s, VMAE=0.69 m/s, VMAPE=29.75%, $\beta \;RMS=25.7^{{}^{\circ}}$, $\beta MAE=16.0^{{}^{\circ}}$. Similarly, the errors were VRMS=1.58 m/s, VMAE=1.14 m/s, VMAPE=44.80%, $\beta \;RMS=39.0^{{}^{\circ}}$, $\beta \;MAE=28.0^{{}^{\circ}}$ in the case of 10-min averaged values and VRMS=0.52 m/s, VMAE=0.41 m/s, VMAPE=24.47%, $\beta \;RMS=44.5^{{}^{\circ}}$, $\beta \;MAE=30.9^{{}^{\circ}}$ in the case of the hourly averaged values.

**Figure 11:**
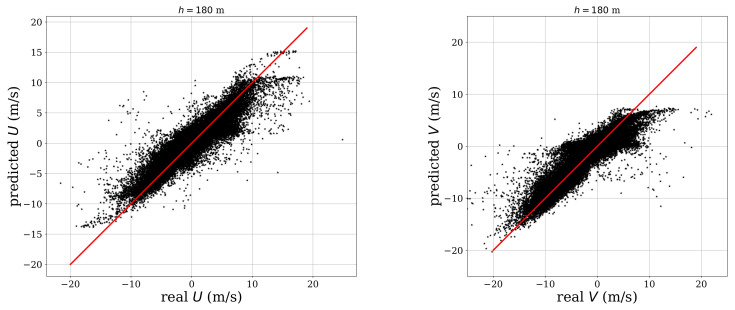
Results for estimation of wind speed at 180 m. **Left image**: *u*, CC=0.90, **right image**: *v*, CC=0.88.

Figure 11 shows the scatter plot of the measured and estimated wind components at h=180 m. The plot shows a constant scatter for all the values of *u* and *v*. The behavior was similar for every height. However, the scatter increased for higher *h* values and diminished for lower ones.

### 4.2. Validation against Alternative Methods

Finally, an additional exercise was done in order to determine if the complexity of the proposed methodology, based on neural networks, made it possible to overperform more conventional approaches. Four other models were used for comparison. The first one estimated the wind speed at certain altitude following the log law [38]:

(15) $$\frac{V_{1}}{V_{2}}=\frac{\log\frac{h_{1}-d}{z_{0}}}{\log\frac{h_{2}-d}{z_{0}}},$$

where the parameter *d* is known as the displacement length and $z_{0}$ as the roughness parameter. We calculated the value of $z_{0}$ and *d* using least squares adjustment to the same measurements that were used to train the neural network. The value of $z_{0}$ is 1.91 m which, as we mentioned before, was typical of regions with a mixture of low-rise and high-rise buildings. It can be assumed also that it was the worst case scenario for boundary layer estimation. Then, method performance would probably improve when more homogeneous terrain was selected.

The second method assumed that the mean profile—the temporal mean of all the observations—was always the best estimation, so it did not consider any ground measurements.

The third was a power law extrapolation given by:

(16) $$V_{1}=V_{0}{\left(\frac{h_{1}}{z_{0}}\right)}^{\alpha },$$

where α is a power-law coefficient determined by the shear between $h_{1}$ and $z_{0}$, and it is also calculated using least squares adjustment.

Finally, another machine learning algorithm was implemented. From previous works, the Random Forest was applied following the method described by Bodini and Optis [26].

**Figure 12:**
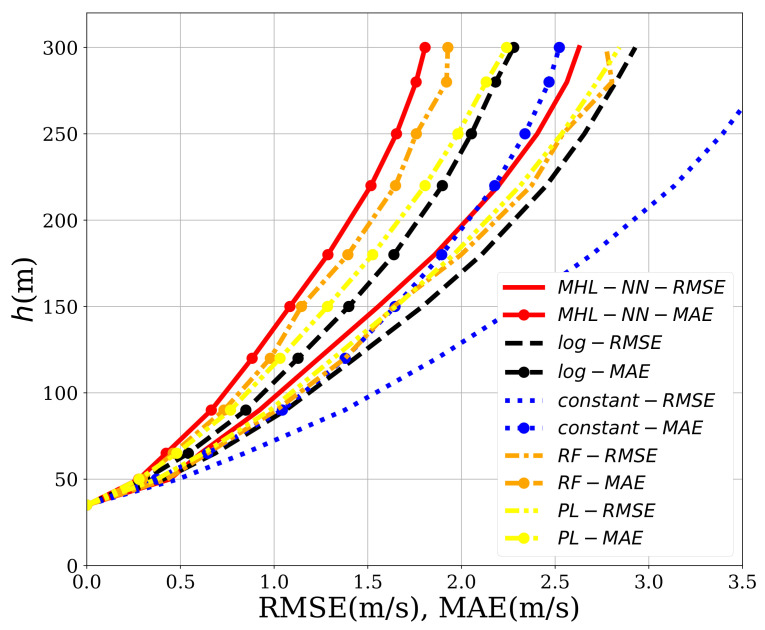
Comparison of the RMSE and MAE for the method described in this work (*MHL-NN*), the log law (*log*), the power law (*PL*), the random forest (*RF*) and the one that estimates always the mean profile (*const*).

The results are shown in Figure 12. The neural network outperformed the others for every height. Even for low heights (up to 90 m), the differences between the three models were smaller, we could find that the reduction in MAE achieved by the MHL-NN method oscillated between the 17% (h=250 m) and 24% (h=90 m) compared to traditional methods. Regarding to RMSE, the reduction yielded between 7% (h=250 m) and 22.5% (h=65 m). The Random Forest algorithm also outperformed the traditional algorithms although to a lesser extent. However, the main advantage of the proposed method was its capability to predict the wind direction (see Figure 9 and Figure 10) for which a simple analytical method could not be found.

The forecasting accuracy was compared against the other method conducting a statistical test known as a Wilcoxon signed-rank test [48,49]. The test results can be seen in Table 8 showing statistical significance.

### 4.3. Flexibility and Growth Capacity

The current study has focused on analyzing the feasibility and advantages of combining lidar sensors and neural networks. Nevertheless, the basic configuration of the neural network can be easily modified to include other relevant effects.

For example, Vassallo, Krishnamurthy and Fernando [23] suggest that the atmospheric stability plays a key role in the estimation of wind speed. Between the different meteorological parameters that influence the atmospheric stability, it can be found the temperature and radiation, which are directly related to the day of the year and time of measurement.

**Figure 13:**
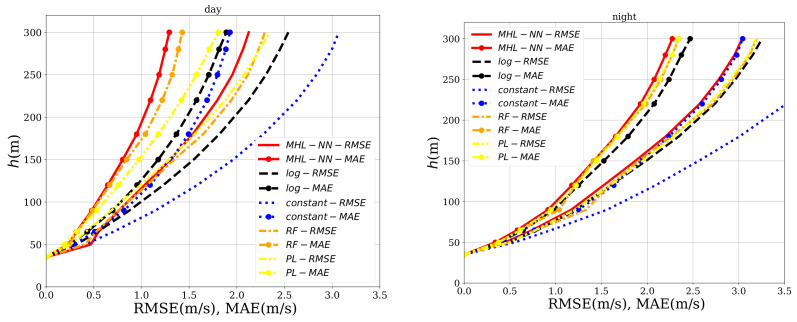
Comparison of the RMSE and MAE for the method described in this work (*MHL-NN*), the log law (*log*), the power law (*PL*), the random forest (*RF*) and the one that estimates always the mean profile (*const*). **Left image**: using only daily measurements. **Right image**: using only nightly measurements.

To study their effects, two new nodes can be added to the input layer of the neural network, one for the hour and one for the day of the measurements. In addition, the data were split into two sets according to whether the measurements were taken during the day (8:00 AM–20:00 PM) or at night (20:00 PM–8:00 AM). Therefore, for each altitude, two neural networks were created: one trained using the hours between 8:00 AM–20:00 PM and one using the interval 20:00 PM–8:00 AM. The results can be seen in Figure 13, in which all the methods are readjusted using the same time intervals. The most obvious finding to emerge from the analysis is that the error was much smaller at day, when the ANN clearly outperformed the other methods. The wind speed RMSE and MAE were further reduced between a 20% and 25% for h>120 m when compared with the error values obtained in Section 4.2. At night, all methods decreased in their performance. Just behind the ANN, the random forest achieved the second-best mark. The results suggested that temperature and radiation played an important role in the wind dynamics of the region for h>90 m.

Another important variable affecting the ABL is precipitation and its associated downdraft [50]. However, in this particular case, including the precipitation data into the neural network did not improve the accuracy of the method. In fact, to check if the interpolation errors were related to the precipitation, the Pearson coefficient between these errors and the amount of precipitation was computed. For the case of the MAE, CC=0.08. Similarly, low values were also found for the RMSE and MAPE. It is worth mentioning that in other locations, the precipitation may play a key role.

## 5. Conclusions

Within this work we constructed a novel methodology to estimate the vertical wind profile of the atmospheric boundary layer that only requires a single point measurement close to the terrain. The methodology makes use of a neural network and its configuration and training process has been evaluated and optimized along the paper. All this work has been possible thanks to the ABL wind data collected by a lidar measurement station located at the Universidad de León (Spain) for up to two years.

In order to have an operational neural network, we firstly analyze the amount of data required for the training process. The conclusion is that selecting 5 consecutive days of observations for each month of the year is the best option. Additionally, the definition of the wind vector seems to play a role in the process. Although the works available in the literature have used a definition based on the modulus of the vector and its direction, the analysis carried out in this work proved that better results can be achieved in the prognosis process if wind vector is defined by its components (north and west). Apart from the training process, the architecture of the neural network has been also defined looking for the optimal performance. It was determined that the optimal architecture for the neural network consists of one hidden layer with 128 nodes. The input layer has 6 nodes, for the last three measurements (u and v wind components) taken at the reference altitude. Finally, the neural network could employ a wide range of optimization algorithms. Some of the most common ones were tested looking for the best option, the RMSprop was selected as it presented the higher accuracy.

Once the neural network was fully defined and trained, it has been able to show it capabilities to estimate ABL wind profiles for altitudes up to 300 m above the ground. When compared with more traditional (but simpler) methods like the logarithm and power laws, it has exhibited a better performance at every height. For example, the mean absolute error (MAE) is reduced between 17% and 24%, depending on the considered height. Furthermore, the proposed neural network is able to also outperform other complex techniques used for wind estimation, such as the random forest method. However, differences are notably smaller in that case (MAE is reduced by about 5%).

Although moving from a quite simple analytical approach (like the logarithm law) to a more complex one (like the one here proposed) requires a certain effort, it is compensated by the higher accuracy of the prognosis process. Additionally, it should be noticed that the notable performance of the logarithm and power laws has been possible thanks to the availability of a large set of wind profile measurements required for the training of the neural network. This made possible to accurately define the terrain aerodynamic roughness.

The neural network architecture of the present study can be used as a blueprint to develop a more complex network. That will make possible to include relevant meteorological parameters such as the date, the time of the day or the precipitation, improving even more the model performance (as envisaged in Section 4.3). Once the information about the time of the day is made available, it is possible to train two different networks: one for the day and another for the night. The analysis of the results has demonstrated that wind estimation process during the day time using neural networks is much more accurate than during the night. This is probably due to the ABL wind flows influenced by the thermal effects during the day. On the other hand, no seasonal effects could be found producing differences between summer and winter period.

The proposed methodology makes possible to optimize the usage of lidar measurement stations, which are usually a very expensive and relatively scarce resource. Thanks to the neural network, the lidar is only needed for the collection of the training data, and it can be replaced after that by a much cheaper local sensor (e.g., a rotating cup anemometer). Thus, it is possible to use a single lidar station to monitor wind profiles at different locations, moving it from one place to another and avoiding the need of a single station for each location.

## Figures and Tables

**Table 1:** Main lidar characteristics. The accuracy is computed as measured against a calibrated moving target.

Range	10–300 m
Height measurements	10 User Configurable
Sampling rate	50 Hz
Wind speed range	1–70 m/s
Accuracy	Wind speed: 0.1 m/s Direction variation: <0.5${}^{{}^{\circ}}$

**Table 2:** Results of the neural network simulations performed to determine the best set of data to train the network. *u* and *v* were used as input variables.

Amount of Data	Selection	uRMSE (m/s)	vRMSE (m/s)	VRMSE (m/s)	βRMSE (rad)	uMAE (m/s)	vMAE (m/s)	VMAE (m/s)	βMAE (rad)	Simulated VRMS (m/s)	Real VRMS (m/s)
Full month	Jan	1.95	2.12	2.27	1.54	1.37	1.51	1.63	0.80	5.02	5.71
	Feb	1.83	2.04	2.12	1.40	1.26	1.48	1.52	0.72	5.05	
	Aug	1.69	2.09	1.98	1.38	1.19	1.47	1.38	0.70	4.70	
	Sept	1.71	2.06	1.93	1.42	1.22	1.45	1.36	0.72	5.01	
5 day/month	1 to 5	1.67	2.00	1.88	1.38	1.17	1.42	1.33	0.70	5.14	5.71
	6 to 10	1.65	2.00	1.89	1.38	1.15	1.43	1.34	0.70	5.10	
Two full months	Jan and Feb	1.78	2.07	2.12	1.42	1.24	1.49	1.51	0.73	5.18	5.71
	Aug and sept	1.69	2.08	1.95	1.39	1.20	1.45	1.36	0.71	4.77	
2 neuronal networks	Jan and Sept	1.75	2.12	2.07	1.44	1.25	1.51	1.46	0.73	5.04	5.71
	Feb and Aug	1.72	2.06	2.01	1.37	1.20	1.47	1.40	0.70	4.84	

**Table 3:** Results of the neural network simulations performed to determine the best set of data to train the network. V and β were used as input variables.

Amount of Data	Selection	uRMSE (m/s)	vRMSE (m/s)	VRMSE (m/s)	βRMSE (rad)	uMAE (m/s)	vMAE (m/s)	VMAE (m/s)	βMAE (rad)	Simulated VRMS (m/s)	Real VRMS (m/s)
Full month	Jan	5.26	3.44	1.93	2.34	3.06	2.17	1.40	1.50	5.69	5.71
	Feb	3.32	2.80	1.83	2.01	2.26	1.93	1.33	1.21	5.34	
	Aug	3.54	2.66	1.88	1.99	2.33	1.85	1.37	1.20	5.06	
	Sept	2.76	2.32	1.86	1.84	1.90	1.77	1.39	1.05	5.25	
5 day/month	1 to 5	2.79	2.44	1.80	1.85	1.86	1.75	1.33	1.05	5.49	5.71
	6 to 10	2.80	2.37	1.78	1.85	1.89	1.76	1.33	1.06	5.39	
Two full months	Jan and Feb	3.60	3.12	1.87	2.02	2.42	2.05	1.38	1.22	5.76	5.71
	Aug and Sept	3.07	2.37	1.84	1.91	2.08	1.74	1.35	1.11	5.20	
2 neuronal networks	Jan and Sept	4.10	2.60	2.36	1.79	1.91	2.04	1.38	1.21	5.38	5.71
	Feb and Aug	3.21	2.50	1.87	1.91	2.12	1.78	1.35	1.12	5.09	

**Table 4:** Results of the neural network simulations performed to determine the optimal architecture.

Nodes in 1º Layer	Nodes in 2º Layer	uRMSE (m/s)	vRMSE (m/s)	VRMSE (m/s)	βRMSE (rad)	uMAE (m/s)	vMAE (m/s)	VMAE (m/s)	βMAE (rad)	Simulated VRMS (m/s)	Real VRMS (m/s)
32	-	1.66	2.02	1.93	1.38	1.15	1.43	1.36	0.71	5.01	5.71
64	-	1.67	2.00	1.88	1.38	1.17	1.42	1.33	0.70	5.14	
128	-	1.65	1.98	1.87	1.37	1.16	1.41	1.31	0.70	5.08	
256	-	1.65	1.99	1.86	1.37	1.15	1.43	1.32	0.70	5.20	
64	8	1.65	1.99	1.91	1.375	1.15	1.41	1.34	0.70	5.00	
128	4	1.67	2.05	2.01	1.39	1.15	1.46	1.4	0.71	4.78	

**Table 5:** Results of the neural network simulations performed to determine the best time period for the input data to use.

Time Period	uRMSE (m/s)	vRMSE (m/s)	VRMSE (m/s)	βRMSE (rad)	uMAE (m/s)	vMAE (m/s)	VMAE (m/s)	βMAE (rad)	Simulated VRMS (m/s)	Real VRMS (m/s)
30 min	1.65	1.98	1.87	1.37	1.16	1.41	1.31	0.70	5.08	5.71
60 min	1.64	2	1.89	1.41	1.17	1.41	1.33	0.72	4.99	
10 min	1.69	2.04	1.93	1.41	1.19	1.46	1.38	0.73	4.10	
3 h	1.65	2	1.95	1.41	1.16	1.43	1.38	0.72	5.20	
6 h	1.67	2.01	1.92	1.42	1.16	1.42	1.34	0.74	4.98	
12 h	1.67	2.04	1.87	1.45	1.17	1.45	1.31	0.75	5.18	

**Table 6:** Results of the neural network simulations performed to choose the best optimization algorithm.

Algorithm	uRMSE (m/s)	vRMSE (m/s)	VRMSE (m/s)	βRMSE (rad)	uMAE (m/s)	vMAE (m/s)	VMAE (m/s)	βMAE (rad)	Simulated VRMS (m/s)	Real VRMS (m/s)
RMSprop	1.65	1.98	1.87	1.37	1.16	1.41	1.31	0.70	5.08	5.71
SGD	1.68	2.02	1.91	1.39	1.18	1.44	1.35	0.70	5.06	
Adam	1.66	1.99	1.90	1.38	1.17	1.41	1.35	0.70	5.20	
Nadam	1.65	2.02	1.91	1.37	1.15	1.42	1.33	0.70	4.92	
Ftrl	1.70	2.03	1.92	1.39	1.19	1.45	1.36	0.71	5.04	

**Table 7:** Performance of the vertical wind profile prognosis process when using the developed neural network.

	RMSE (m/s)		MAE (m/s)		MAPE (%)		Pearson Coefficient	
Objective h (m)	u	v	u	v	u	v	u	v
50	0.45	0.45	0.29	0.29	13.06	14.94	0.99	0.99
65	0.59	0.54	0.43	0.43	18.44	20.41	0.98	0.98
90	0.92	0.86	0.68	0.68	26.07	32.48	0.96	0.96
120	1.19	1.28	0.86	0.86	30.28	43.50	0.94	0.93
150	1.43	1.66	1.02	1.02	35.54	49.67	0.92	0.90
180	1.66	2.01	1.16	1.16	40.08	55.34	0.90	0.88
220	1.94	2.36	1.32	1.32	43.34	58.78	0.87	0.86
250	2.10	2.56	1.43	1.43	46.20	62.11	0.85	0.84
280	2.25	2.72	1.51	1.51	47.98	63.61	0.84	0.83
300	2.28	2.75	1.51	1.51	46.62	63.03	0.84	0.83

**Table 8:** Wilcoxon signed-rank test. N=1000. One-sided test, with hypothesis that the errors of ANN are smaller compared against the other methods.

Model	Statistic	*p* Value
ANN vs. Random Forest	238,018	0.09
ANN vs. Power Law	100,668	$10^{-6}$
ANN vs. Constant Model	110,970	$10^{-6}$
ANN vs. Logarithmic Law	233,676	0.03

## Abbreviations

The following abbreviations are used in this manuscript:

*a*	Sound speed
ABL	Atmospheric boundary layer
AGL	Above ground level
ANN	Artificial neural networks
ATM	Air traffic management
CC	Pearson correlation coefficient
*d*	Displacement length
DBS	Doppler beam swinging
*h*	Height
$K_{xy}$	Temporal cross-covariance
lidar	Light Detection and Ranging
MAE	Mean absolute error
MAPE	Mean absolute percentage error
MHL-NN	Multi-hidden layer neural networks
RMS	Root mean square
RMSE	Root mean square error
SSE	Sum of squares of the network errors
*u*	North wind component
*v*	West wind components
V	Wind speed modulus
$z_{0}$	Roughness parameter
β	Wind speed direction
σ	Mapping function
